# Mental Health Mobile Apps: From Infusion to Diffusion in the Mental Health Social System

**DOI:** 10.2196/mental.3954

**Published:** 2015-03-31

**Authors:** Marlene Lynette East, Byron C Havard

**Affiliations:** ^1^ East Counseling Services, Inc. Private Counseling Practice Panama City Beach, FL United States; ^2^ The University of West Florida Instructional & Performance Technology Specialization Pensacola, FL United States; ^3^ The University of West Florida Associate Professor, Instructional & Performance Technology Department Pensacola, FL United States

**Keywords:** clinical efficacy, counselors, ethical codes, innovation diffusion, instructional technology, mental health, mHealth, mobile health, smartphone

## Abstract

The roles of mental health educators and professionals in the diffusion of mental health mobile apps are addressed in this viewpoint article. Mental health mobile apps are emerging technologies that fit under the broad heading of mobile health (mHealth). mHealth, encompassed within electronic health (eHealth), reflects the use of mobile devices for the practice of public health. Well-designed mental health mobile apps that present content in interactive, engaging, and stimulating ways can promote cognitive learning, personal growth, and mental health enhancement. As key influencers in the mental health social system, counselor educators and professional associations may either help or hinder diffusion of beneficial mHealth technologies. As mental health mobile apps move towards ubiquity, research will continue to be conducted. The studies published thus far, combined with the potential of mental health mobile apps for learning and personal growth, offer enough evidence to compel mental health professionals to infuse these technologies into education and practice. Counselor educators and professional associations must use their influential leadership roles to train students and practitioners in how to research, evaluate, and integrate mental health mobile apps into practice. The objectives of this article are to (1) increase awareness of mHealth and mental health mobile apps, (2) demonstrate the potential for continued growth in mental health mobile apps based on technology use and acceptance theory, mHealth organizational initiatives, and evidence about how humans learn, (3) discuss evidence-based benefits of mental health mobile apps, (4) examine the current state of mHealth diffusion in the mental health profession, and (5) offer solutions for impelling innovation diffusion by infusing mental health mobile apps into education, training, and clinical settings. This discussion has implications for counselor educators, mental health practitioners, associations, continuing education providers, and app developers.

## Introduction

This article addresses the roles of mental health educators and professionals in the diffusion of mental health mobile apps. These emerging technologies fit under the broad heading of mobile health (mHealth), where mHealth, encompassed within electronic health (eHealth), reflects the use of mobile devices for the practice of public health. The objectives are to (1) increase awareness of mHealth and mental health mobile apps, (2) demonstrate the potential for continued growth in mental health mobile apps based on technology use and acceptance theory, mHealth organizational initiatives, and evidence about how humans learn, (3) discuss evidence-based benefits of mental health mobile apps, (4) examine the current state of mHealth diffusion in the mental health profession, and (5) offer solutions for impelling innovation diffusion by infusing mental health mobile apps into education, training, and clinical settings. This discussion has implications for counselor educators, mental health practitioners, associations, continuing education providers, and app developers.

## The Mobile Health Explosion

### Overview

**Figure 1:**
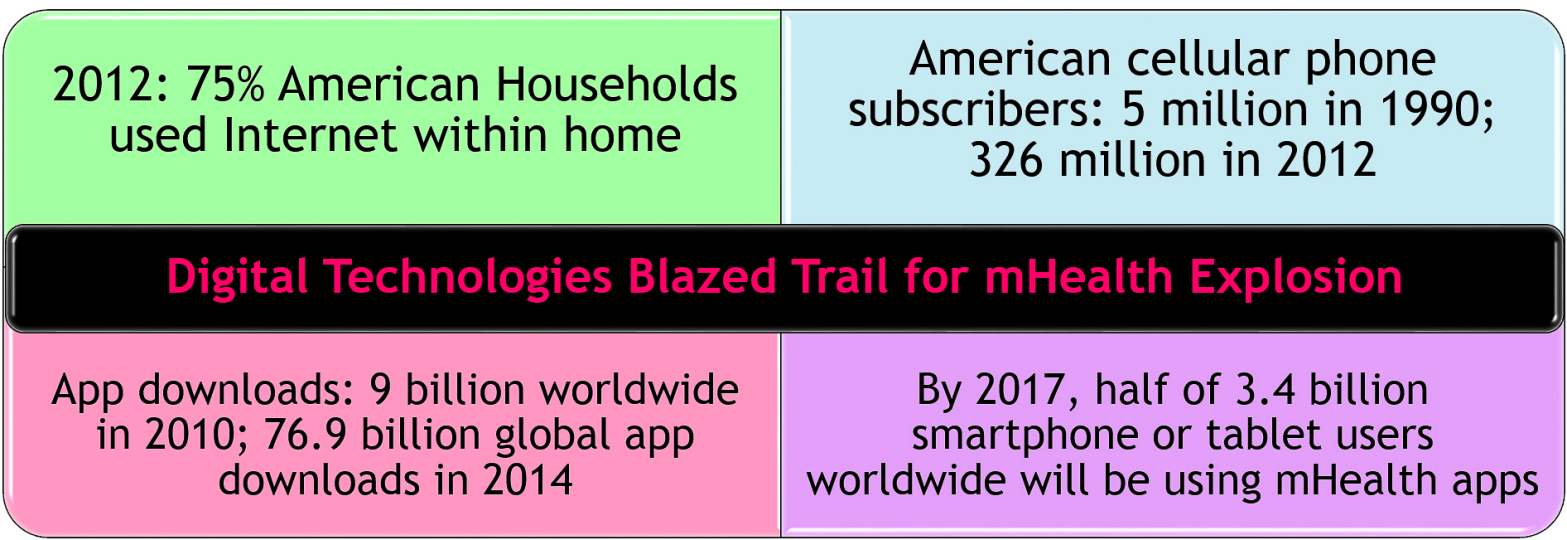
Digital technologies blazed the trail for the mHealth explosion.

Nascent digital technologies blazed the trail for the explosion of mHealth and mobile health apps (Figure 1). In 2012, 75% of American households used the Internet within the home [1], and 79% of Americans accessed the Internet during the prior month [2]. Some form of broadband is available to almost 99% of the US population [1]. Cellular phone subscribers grew from approximately 5 million Americans in 1990 to over 326 million in 2012 [3]. As of October 2012, 88% of Americans ages 25 and older used mobile phones [1].

According to ABI Research, 9 billion apps were downloaded worldwide to mobile phones in 2010. In 2011, there were roughly 17,000 health-related applications for iPhones, Android-based devices, and other mobile phones and tablets [4]. Of the 9,000 consumer health apps available in 2011, approximately 6% related to mental health, 11% to stress management, 4% to sleep, and 2% to smoking cessation [5]. International Data Corporation had forecasted 76.9 billion global app downloads for 2014 [6], where the mHealth market accounted for nearly $9 billion [7]. By 2017, it is estimated that half of the 3.4 billion mobile phone or tablet users worldwide will be using mHealth apps [8].

### Potential for Mental Health Mobile Apps Growth

**Figure 2:**
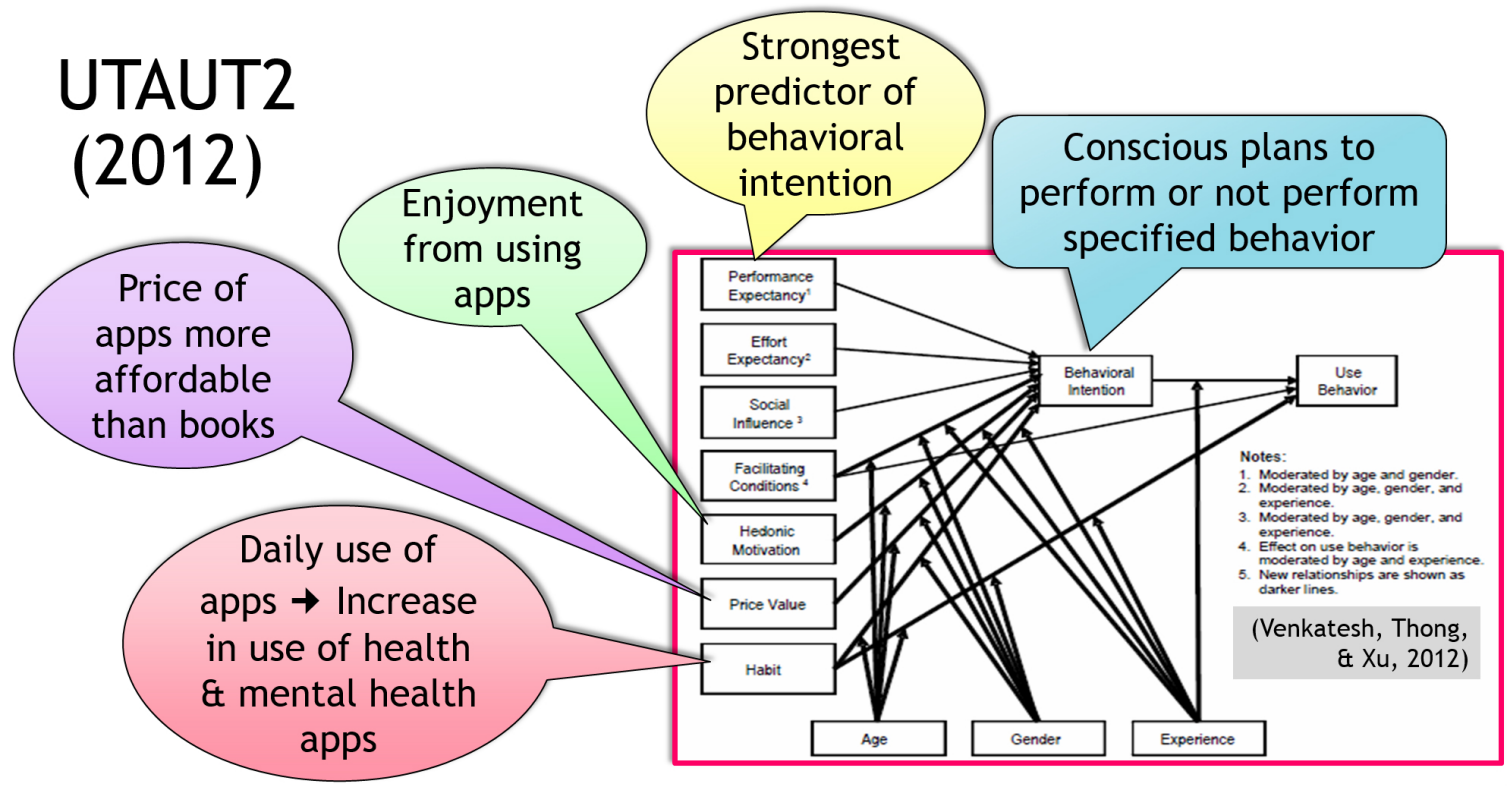
Potential for diffusion of mental health mobile apps based on the unified theory of use & acceptance 2 (UTAUT2). © 2012, Regents of the University of Minnesota, used with permission.

Evaluating mental health mobile app development and use through the lens of the unified theory of acceptance and use of technology (UTAUT) provides insight into the prospective continued growth of mHealth technologies. In 2012, Venkatesh and colleagues applied UTAUT to consumers and developed UTAUT2 (Figure 2) [9]. As app developers apply the findings from this comprehensive technology use and acceptance theory that pulled together decades of research, it follows that numbers of apps and the number of app users will continue to increase. The constructs identified in UTAUT2 may be applied specifically to a consumer’s behavioral intention to use mental health mobile apps.

### Performance Expectancy

Performance expectancy is the degree to which a mental health mobile health app will provide benefits to consumers performing certain activities. According to Venkatesh et al, this construct has been shown to be the strongest predictor of behavioral intention [9]. When mental health providers endorse and encourage the use of particular apps, consumer performance expectancy is heightened, increasing the likelihood that patients will use provider-recommended apps.

### Effort Expectancy

Effort expectancy is the degree of ease associated with consumers’ use of mental health mobile apps. With the passage of time, consumers become more experienced with mobile apps and understand how easy it is to use a personal phone to interact with mental health information. Consumers and practitioners will soon learn that very little effort and time is required to benefit from using mental health mobile apps.

### Social Influence

Social influence is the extent to which consumers perceive that family and friends believe mental health mobile apps should be used. As the number of app downloads increases, so too will the social influence on even more people to use mental health mobile apps. Mental health professionals may also exert social influence on colleagues and patients to use mental health mobile apps.

### Facilitating Conditions

Facilitating conditions refers to consumers’ perceptions of the resources and support available to perform a behavior. As consumers become adept in using mobile phone features and interacting with apps, the ability to connect to the Internet becomes more ubiquitous, and as app developers offer more user-friendly, evidence-based apps and technical support, conditions will be facilitated to increase app use.

### Hedonic Motivation

Hedonic motivation, enjoyment from using apps, is also a predictor of intention to use apps. Using mobile apps to access and interact with mental health information can be much more engaging and stimulating than sources of mental health information used in the past.

### Price Value

Price value is an important factor as consumers have to bear the costs associated with purchasing mobile apps. Many mental health mobile apps are less than $5.00 and many are free. The price value of apps is much more affordable than the price of many books or workbooks.

### Habit

Habit is the extent to which people tend to perform behaviors automatically because of learning. “Habit is a perpetual construct that reflects the results of prior experiences” (p161) [9]. As people increasingly use a variety of apps as a daily habit, this will increase the likelihood that people will also use mental health mobile apps.

## National and Global Mobile Health Initiatives

### Overview

**Figure 3:**
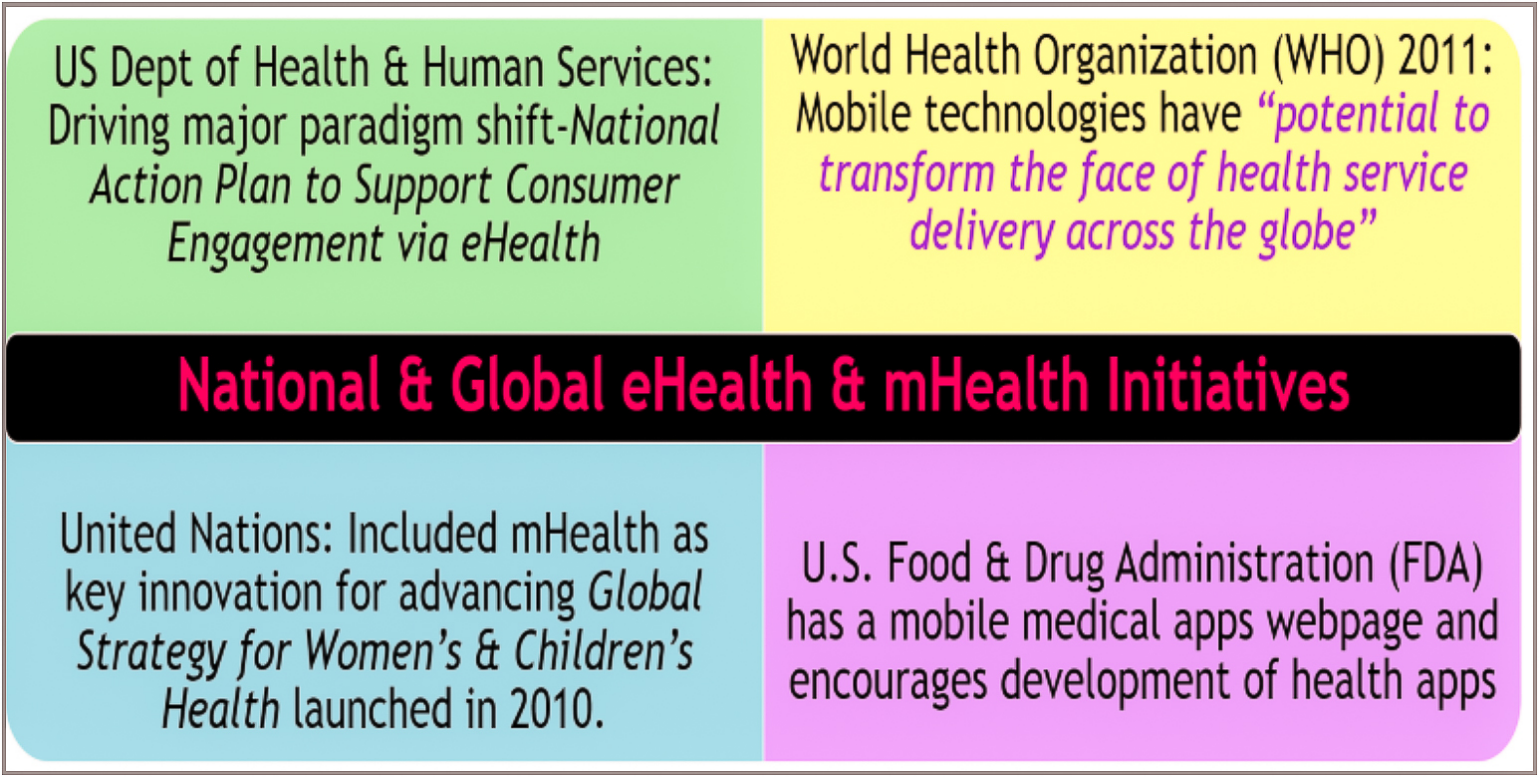
National and global electronic and mobile health initiatives.

Grasping the potential of mHealth, national and global level organizations are also lighting the way with initiatives for a healthcare paradigm shift and the mHealth explosion (Figure 3). In 2013, the US Department of Health and Human Services Office of the National Coordinator for Health Information Technology released the National Action Plan to Support Consumer Engagement via eHealth [10]. The goal is to increase eHealth information access, support the development of related digital tools, and shift attitudes from traditional patient-provider roles to patient-centered care.

The World Health Organization (WHO) stated that mobile technologies have “potential to transform the face of health service delivery across the globe.” (p1) [11] WHO’s mHealth report indicates applications of mobile phone technologies in maternal and child health, diseases, access to emergency and general health services, and treatment enhancement. The WHO mHealth report also noted the United Nations included mHealth as a key innovation for advancing the Global Strategy for Women’s and Children’s Health, launched in New York in 2010 [11]. Another report by the WHO, the Mental Health Action Plan 2013-2020, includes a goal to promote self-care through the use of mobile health technologies [12].

In addition, the US Food and Drug Administration (FDA) features a mobile medical apps page on its website and states “the widespread adoption and use of mobile technologies is opening new and innovative ways to improve health and health care delivery” and “these tools are being adopted almost as quickly as they can be developed.” The FDA website indicates the agency encourages development of mobile medical apps that improve health care and provide health information [13].

### Capabilities of Mental Health Mobile Apps

**Figure 4:**
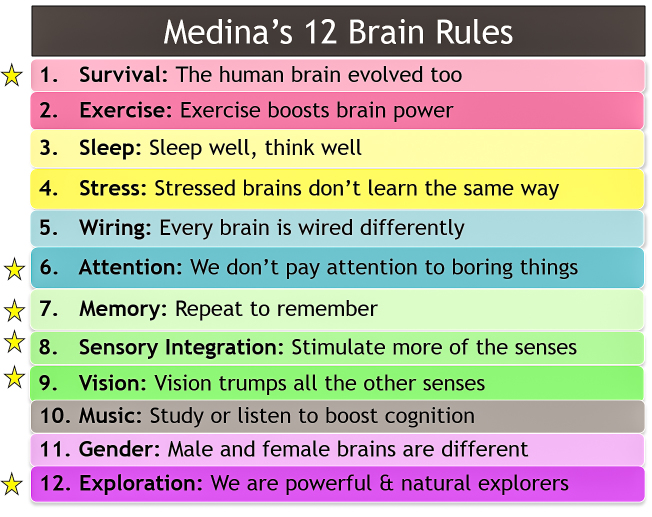
Six of Medina’s 12 Brain Rules that relate well to the potential of mental health mobile apps to enhance learning and personal growth. From Brain Rules, by John Medina. Copyright ©2008 by John Medina, used with permission.

In his book, *Brain Rules, 12 Principles for Surviving and Thriving at Work, Home, and School,* molecular biologist John Medina conveyed scientific evidence about how brains learn and work [14]. The studies that formed a basis for Medina’s *12 Brain Rules* (Figure 4) were published in peer-reviewed journals and successfully replicated. Some of Medina’s brain rules relate well to the capabilities of mental health mobile apps to enhance mental health intervention and treatment.

### Rule #1 Survival: the Human Brain Evolved Too

With technology interaction, the human brain evolves. Former homework tools such as handouts and workbooks are outdated. As people continue to embrace mobile phone technologies, people will likely be less interested in writing on handouts.

### Rule #6 Attention: We Don’t Pay Attention to Boring Things

Well-designed mobile apps are much more interesting than black words on a white sheet of paper. App designers apply research about colors, fonts, backgrounds, and learning activities. Applying this research to app content helps engage attention and maintain the interests of users.

### Rule #7 Memory: Repeat to remember

Working memory, also known as short-term memory, is limited in capacity. mHealth apps can help people learn new information to improve mental health. It is easy to tap and slide on a mobile phone screen. The built-in features of mobile apps permit frequent repeating of information; repeating facts engages the transfer of information to long-term memory. In addition, mobile phones with app capabilities permit users to access limitless sources of information on the Internet in a readily-accessible personal handheld device, enhancing the abilities of users to learn and apply health information.

### Rule #8 Sensory Integration: Stimulate more of the senses

With colorful graphics, videos, music, audio, and kinesthetic interaction, well-designed apps simultaneously engage several senses; this enhances the learning experience. The study on deep breathing apps by Chittaro and Sioni is an example of sensory integration in a mental health mobile app [15] where the integration of visualization with an historically auditory intervention improved the effectiveness of deep breathing training.

### Rule #9 Vision: Vision Trumps All the Other Senses

Being able to view graphics, diagrams, charts, and videos on mobile phones offers varied learning experiences. Visual explanations of mental health information provide users with the opportunity to deepen their understanding of mental health concerns in an engaging manner.

### Rule #12 Exploration: We Are Powerful and Natural Explorers

Well-designed mental health mobile apps permit users to engage in interactive exploration. Medina notes that humans learn by observing, testing, and experimenting.

Mental health mobile apps offer a framework for implementing what Medina’s research revealed about how to help people learn. Well-designed mental health mobile apps that present content in interactive, engaging, and multisensory approaches can promote cognitive learning, personal growth, and mental health enhancement.

## Evidence-Based Benefits of Mental Health Mobile Apps

Because mental health professionals have an ethical duty to use evidence-based interventions [16-19], it is necessary to evaluate research evidence. Studies regarding efficacy evidence of mental health mobile apps are appearing in journals. Research may be found regarding apps to support treatment for anxiety, depression, social phobia, obsessive compulsive disorder, and posttraumatic stress disorder. Treatments such as cognitive behavior therapy, dialectical behavior therapy, and prolonged exposure therapy are being assimilated into apps that supplement the therapy process. Apps are being used to assist with social work, client monitoring, and psychoeducation.

Luxton et al [20] provided an overview of mobile phone use in behavioral health care. The researchers presented a table of behavioral health apps based on clinical area, platform, and purpose. It is clear from this review that mobile phones are being used to deliver treatment inventions, psychoeducation, audio-recording of sessions, and virtual coaching. The researchers note:

The rapid growth in the use of smartphones has opened a new world of opportunities for use in behavioral health care. Mobile phone software applications (apps) are available for a variety of useful tasks to include symptom assessment, psychoeducation, resource location, and tracking of treatment progress. The latest two-way communication functionality of smartphones also brings new capabilities for telemental health.

p505, [20]

**Figure 5:**
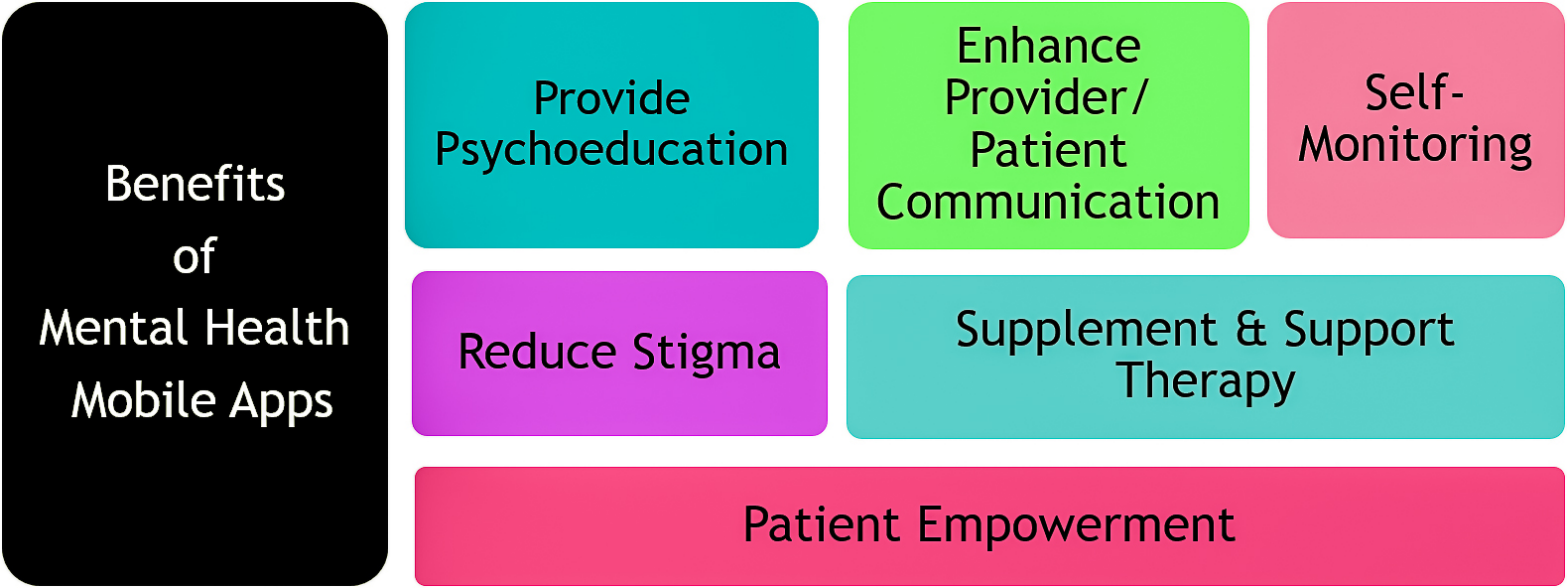
Benefits and uses of mental health mobile apps.

The Luxton et al overview indicates the potential of mental health mobile apps for consumer empowerment, reduction of stigma associated with seeking mental health treatment, self-monitoring, improved patient/provider communication, and enhancement of psychological services (Figure 5). They also highlighted another promising use of mobile phones in behavioral health: external hardware devices such as biofeedback sensors may be connected to mobile phones. Such external devices combined with mobile phone capabilities and app features may be used to improve mental health. In projecting the future of behavioral health digital technology, Luxton et al note:

Future widespread use of smartphone technology in the behavioral health field can be expected. Our increasingly mobile, tech-savvy, and health conscious society will demand care delivery solutions that expand beyond traditional office-based requirements to better fit diverse needs and lifestyles.

p510, [20]

Georgia State University researchers Jabeley and colleagues researched an augmented version of SafeCare, as an in-home child safety mobile phone app intervention [21]. Parents were trained to use a mobile phone (iPhone) to video rooms in their homes to assist in identifying child safety hazards and toxins. The parents shared the videos with an observer in a graduate public health education program and both the observer and the parents used mobile phones to communicate feedback, logistics, and safety content. The researchers found that hazards were reduced across rooms and across participants. As well, the face-to-face time of the home observers was progressively reduced and replaced by video data collection. This study suggests that mobile phones with app capabilities are promising for data collection and for augmenting face-to-face interactions.

In Finland, Lappalainen et al conducted a pilot study to assess the feasibility of the P4Well intervention in treatment of stress-related psychological problems in males [22]. P4Well is a novel intervention which combines modern psychotherapy, cognitive behavioral therapy, and acceptance and commitment therapy, with personal health technologies to deliver the intervention via multiple channels. The intervention includes group meetings, an internet portal, mobile phone applications, and personal monitoring devices.

*Our results confirm the feasibility of the intervention and suggest that it had positive effects on psychological symptoms, self-rated health, and self-rated working ability. The intervention seemed to have a positive impact on certain aspects of burnout and job strain, such as cynicism and over-commitment.* (p1) [22]

Mobile mind mapping apps can support counseling frameworks, such as rational emotive behavior therapy (REBT) as discussed by Warren [23]. A major goal of REBT is to encourage clients to become their own therapists. Clients may use mobile mind mapping apps to implement REBT principles during or immediately after adverse situations, and may use it daily for storing and readily accessing a database of rational thoughts.

Several mobile apps are available to guide users through deep breathing exercises, a technique often used to reduce stress, anxiety, and depression. Audio breathing guides have been the status quo. Mobile apps, however, have presented the ability to add a visual element to deep breathing guides. Chittaro and Sioni conducted an analysis of three approaches to deep breathing training [15]. One employed a traditional audio method, and the other two employed different types of visualizations used together with audio instruction. The researchers analyzed participants’ physiological reactions and subjective perceptions and their findings indicate that visualization can contribute to the effectiveness of breathing training apps.

In addition to the benefits offered by mental health mobile apps, it is evident that target populations are interested in using their phones to monitor and improve their mental health. For example, a study by Torous and colleagues indicated psychiatric outpatients are interested in using their mobile phones to monitor their mental health [24]. The researchers surveyed 320 psychiatric outpatients from clinics in four states and found that about 70% of patients were interested in using mobile phones to monitor symptoms. Based on their results, the researchers concluded there may be fewer patient obstacles to mobile phone application-based clinical monitoring and treatment protocols than commonly thought.

## The Diffusion of Mental Health Mobile Apps in the Mental Health Profession

### Overview

Rogers described innovation diffusion as the process of communicating an innovation over time among members of a social system [25]. Counselor educators and professional associations are key influencers of the diffusion or lack of diffusion of mHealth throughout the mental health social system. Counselor educators and associations must consider the importance of their roles in providing instruction regarding mental health mobile apps and their roles in the innovation diffusion process.

### Counselor Education

Counselor educators are to provide instruction on the most current knowledge, empirically based techniques, procedures, and modalities. In addition, counselor educators are obligated to heed accreditation standards that require education programs to produce evidence of the use and infusion of technology [18]. A technology-infused learning environment is one in which technology permeates the educational setting and the curriculum. Counseling professionals are to actively pursue understanding of how to use technology resources to better serve clients [16,18,19]. Based on these ethical mandates and standards, it is clear that counselor educators are directed to learn about and teach students how to integrate evidence-based mental health mobile apps into practice.

The mental health profession, however, has lagged in technology adoption for counselor education and treatment interventions [20,26]. In an article on the use of social media in counselor education, for example, Tilman and colleagues indicate “The digital age marches on with or without counselor education programs being prepared.” (p1) [27]

Professional Association Conferences

Continuing education for mental health professionals is to reflect current knowledge and emerging developments [17,19], and counselors are to be open to and obtain training on new procedures [16]. Educators and practicing mental health professionals often rely on professional conferences to obtain required continuing education credits. The authors of this article conducted a content analysis in early February 2015 to identify mental health technology-related topics offered at conferences (Table 1).

Thousands of mental health mobile apps are currently available in app stores. However, out of 4,404 session topics at the 12 conferences included in the content analysis, 4.1% (179/4,404) were technology-related topics. Only 0.3% (14/4,404) of topics specifically addressed mental health mobile apps. This analysis of professional association conference topics indicates training is lagging behind development. There is room to include more mental health technology topics at professional conferences. Mental health associations have a timely opportunity to encourage and offer more research and training regarding the use of mental health mobile apps and other mental health technologies.

**Table 1:** Content analysis of mental health professional conferences 2013-2015: technology-related session topics.

Conference	Year^a^	Technology-related topics^b^, n	Total sessions^c^, n	Technology - related topics^d^, %	Mental health mobile app topics^e^, n
American Association for Marriage & Family Therapy	2013	5	161	3.0%	0
American Association for Marriage & Family Therapy	2014	3	77	3.8%	0
American Board of Professional Psychology	2014	0	26	0.0%	0
American Board of Professional Psychology	2015	0	19	0.0%	0
American Counseling Association	2014	20	361	5.5%	0
American Counseling Association	2015	17	457	3.7%	1
American Mental Health Counselors Association	2013	3	82	4.0%	0
American Psychiatric Association	2013	11	512	2.1%	2
American Psychiatric Association	2014	18	597	3.0%	1
American Psychological Association	2013	54	990	5.5%	7
American Psychological Association	2014	43	983	4.4%	3
National Association of Social Workers	2014	5	139	3.5%	0
Total, N or mean (%)		179	4,404	4.1%	14

^a^ This content analysis was completed in early February 2014. Only two conferences had complete conference programs available for 2015 at that time.

^b^Search terms used: app/apps, blog/blogging, digital, distance, eHealth/e-Health, ethics, games/gaming, innovative/innovation, internet, mHealth/m-Health, mobile, online, psychotechnology, social media, technology, telecoaching, teleconsulting, telehealth, telepsychology, twitter, virtual/virtual reality, and web/website/web-based

^c^ Included were conference program courses, case conferences, advances, symposia, lectures, reports, workshops, and forums. Poster sessions were not included due to overlap with other sessions.

^d^ Calculated by dividing technology-related topics by total session number.

^e^Included in technology-related topics

## Solutions for Impelling Diffusion of Mental Health Mobile Apps

### Overview

Mental health mobile apps are considered to be emerging technologies. As mental health mobile apps move toward ubiquity, research will continue to be conducted. The studies published thus far, combined with the potential of mental health mobile apps for learning and personal growth, offer enough evidence to compel mental health professionals to integrate these technologies into education and practice. Counselor educators and professional associations must use their influential leadership roles to train students and practitioners in how to research, evaluate, and integrate mental health mobile apps into practice.

**Figure 6:**
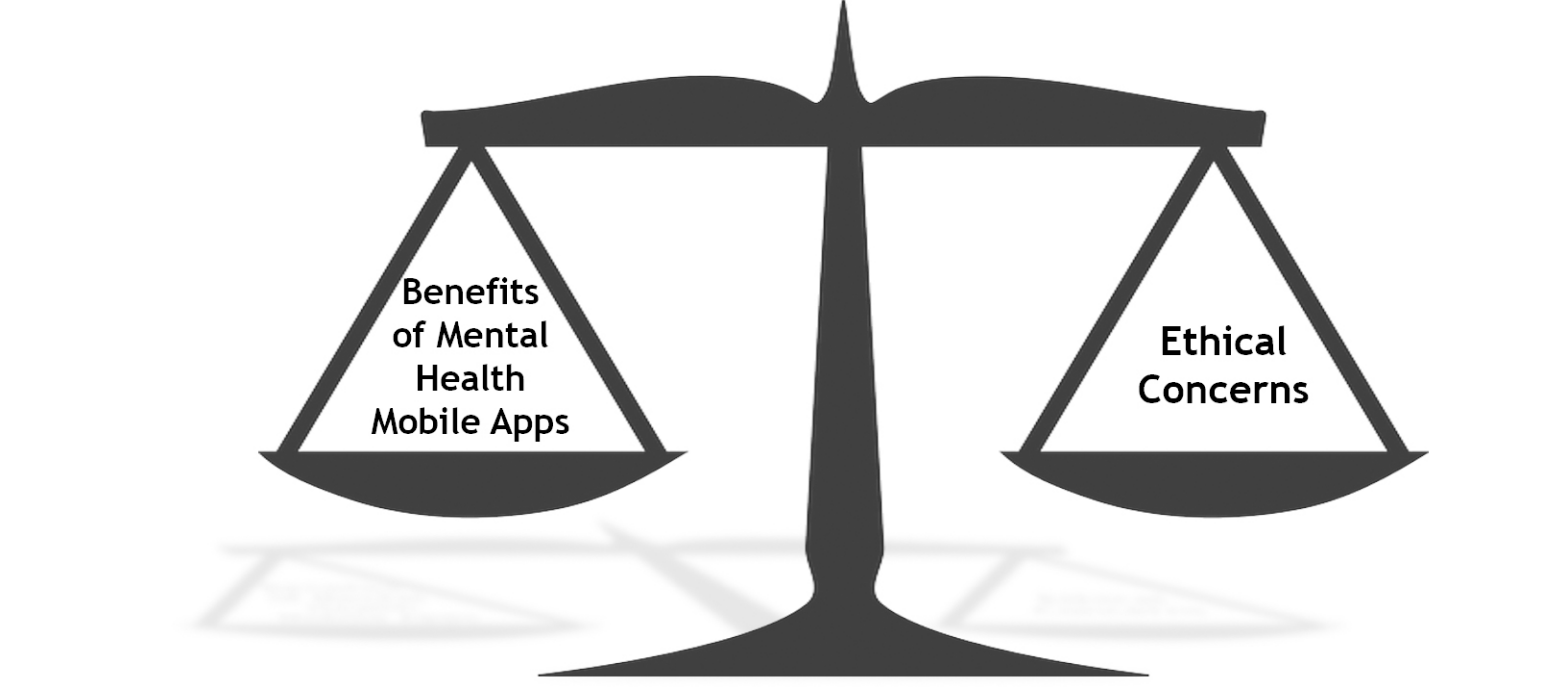
Mental health professionals are to balance ethical concerns with the benefits of mental health mobile apps.

The ultimate guide for using or teaching any therapeutic intervention or mental health technology is to only do what is good for patients and not harm patients. These principles of beneficence and non-maleficence guide ethical codes, laws, and daily practices. In recommending particular mental health mobile apps to patients, professionals must carefully consider issues such as privacy, informed consent, security of confidential data, and potential harm. Since ethical concerns are highly relevant and are not to be ignored, the goal of mental health professionals should be to exercise ethical prudence while simultaneously embracing the benefits of mental health mobile apps (Figure 6).

### Promoting Technological Literacy

Promoting technological literacy will assist counselor educators and professional associations in diffusing the innovation of mental health mobile apps throughout the mental health social system. In the book *Using Technology to Improve Counseling Practice, A Primer for the 21^st^ Century,* Tyler and Sabella synthesized several explanations of technological literacy into the following definition:

*Technological literacy is the intellectual processes, abilities, and dispositions needed for counselors to understand the link among technology, themselves, their clients, and a diverse society so that they may extend human abilities to satisfy human needs and wants for themselves and others.* (p5)[28]

**Figure 7:**
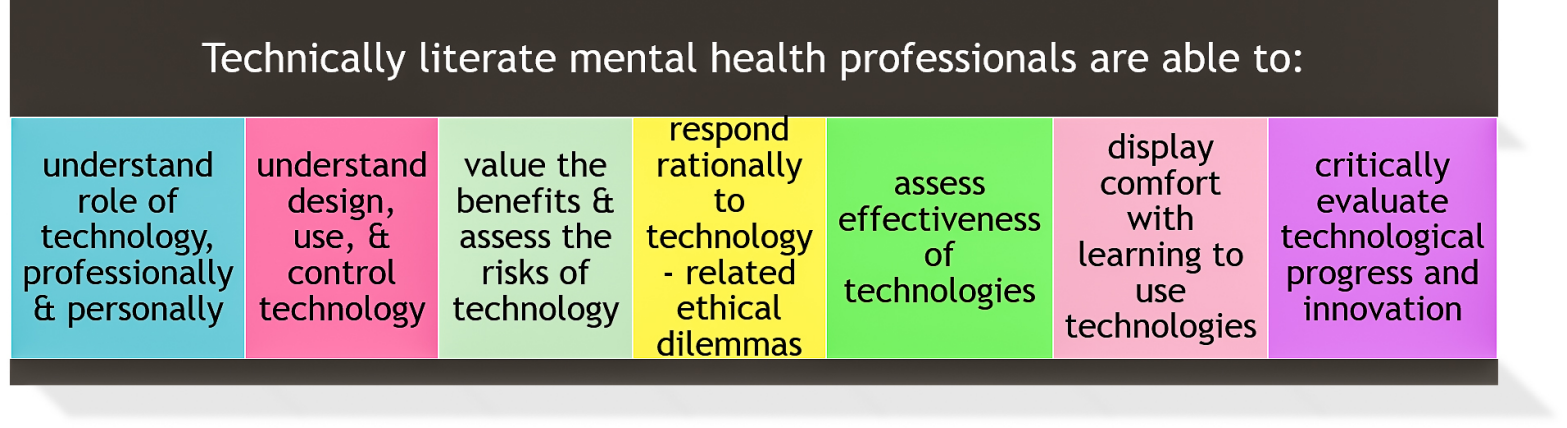
Abilities of technically literate health professionals. From Using Technology to Improve Counseling Practice, A Primer for the 21st Century [29].

Although Tyler and Sabella’s definition was written a decade ago, well before the emergence of mental health mobile apps, it continues to be a useful definition. The authors further define the abilities of technically literate health professionals which are summarized in Figure 7.

**Figure 8:**
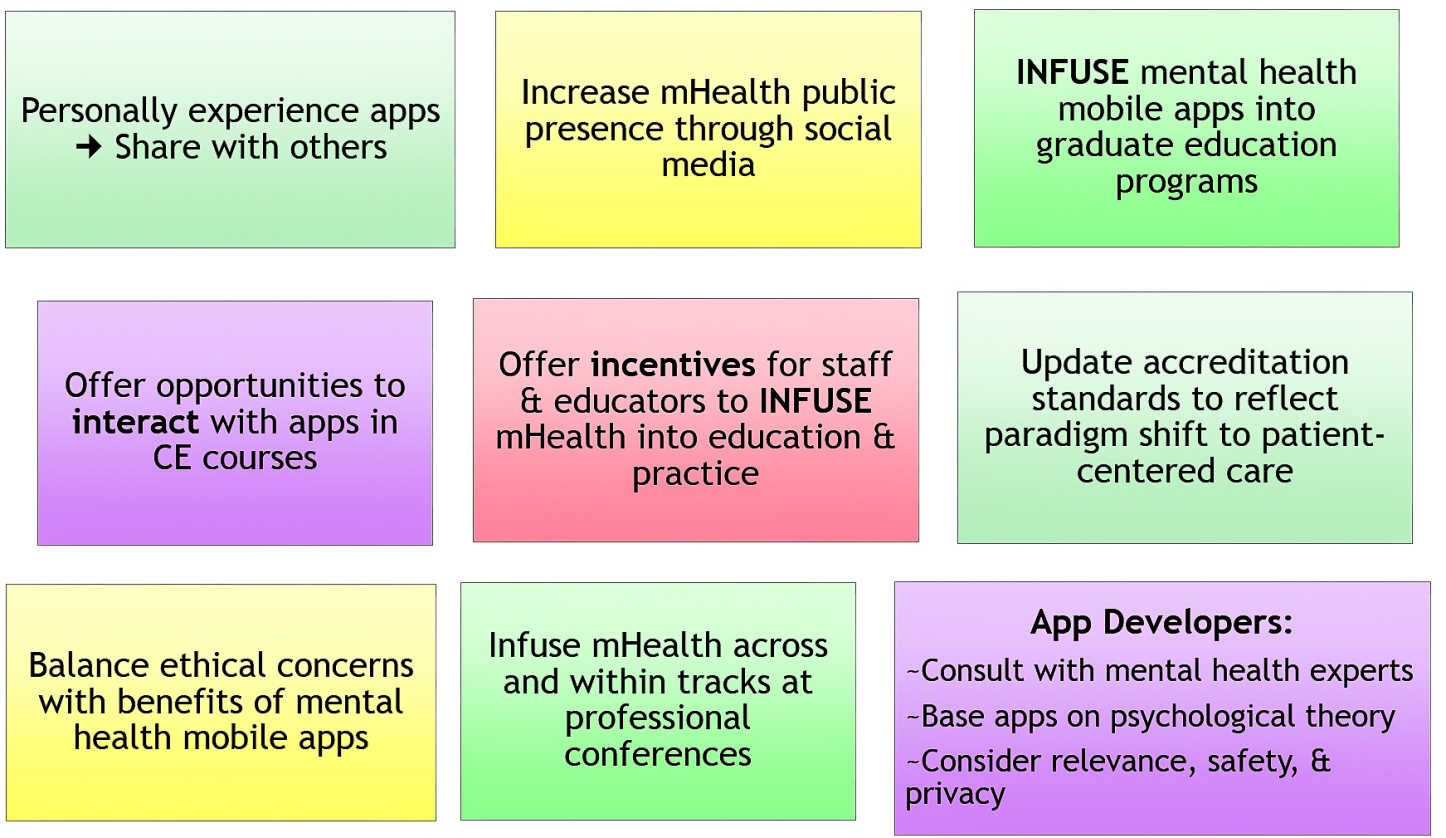
Solutions for impelling diffusion of mental health mobile apps.

### Personally Use Apps

A relevant 14^th^ century proverb is *the proof of the pudding is in the eating.* In innovation diffusion theory, trialability [25] means one must personally experience an innovation in order to fully test that innovation. Professionals may enhance their personal lives with evidence-based exercise and fitness apps, relaxation apps, sleep apps, burnout prevention apps, and continuing education tracking apps. As mental health professionals become more adept in personally using apps, they will be more likely to socially influence colleagues and students to use apps. Together, this will contribute to the diffusion of mental health mobile apps throughout the mental health social system.

### Raise Awareness of Evidence-Based Apps

Practitioners may aim to increase the awareness of mental health mobile apps through social media channels. Websites may be updated to include links to research regarding evidenced-based apps and to include tips for using particular apps. Blogging, micro-blogging (eg, Twitter), and posting on Facebook are means of establishing an online presence regarding evidence-based mental health mobile apps. To increase personal mHealth awareness, practitioners will benefit from following the blogs and postings of other mHealth organizations and professionals.

### Infuse Mental Health Mobile Apps into Graduate Counselor Education

Infusing educational content with mental health mobile apps across and within courses is required in order to develop technologically competent, culturally-relevant mental health professionals. However, accomplishing infusion will be a challenge considering so many university faculty members are digital immigrants [29] who may be reluctant to embrace new technologies [26]. To overcome this problem, counseling departments need to offer professional development workshops regarding mental health mobile apps. These classes need to include hands-on interactivity (trialability) with apps and guidance for infusing these technologies into educational and clinical settings. Offering incentives to encourage effective infusion of mHealth into curricula will enhance technology adoption [25]. A few typically-required graduate-level counseling courses and ideas for learning activities offered for each course are listed in Textbox 1.

**Course and learning activities for infusing mental health mobile apps into graduate level counselor education courses.**

Counseling skills

- Identify and review apps for recognizing and rating intensity of feelings.
- Use a free app builder program to create a counseling skills educational app on a topic such as basic attending skills, empathy, transference and countertransference, stages of counseling interviews, confrontation, interpretation, or multicultural perspectives.

Counseling theories and techniques

- Identify and review apps that indicate a particular theoretical foundation. Write a paper discussing the degree to which the app aligns with that theory.
- Work in groups and assess selected apps in view of multicultural awareness.

Ethics

- Identify ethics codes that are pertinent to the design, development, and therapeutic integration of mental health mobile apps.
- Establish a class webpage for app reviews, and require students to post reviews of a designated number of mental health mobile apps in regards to potential ethical issues.

Individual analysis

- Identify apps with built-in assessments and rating scales, and review and report on the efficacy evidence of these assessments.
- Use a free app builder to design a psychoeducational app on a particular mental health condition.

Life span development

- Identify and review apps that are useful for various developmental stages such as pregnancy, parenting skills, child mental disorders, career development, relationships, disabilities, and mental health enhancement for people who are elderly.
- Work in a group and create a gaming app using a free gaming app creator, and target a particular age group and developmental issue.

Professional issues

- Critique apps promoting an inpatient program and an outpatient counseling center, and describe the strengths and indicate any needs for improvement.
- Create a hypothetical informational app for a counseling center. Address boundaries, confidentiality, abuse reporting, records, fees, roles and responsibilities, referrals, and termination.

Psychological change strategies

- Use a cognitive behavior therapy app throughout the semester, and give a presentation that reflects impressions of the app and how it may be used in therapy.
- Present a hypothetical case study report outlining the integration of an app into the therapeutic process.

Research methods

- Write a research paper critiquing the efficacy evidence for two apps that are promoted as interventions for a particular mental health condition.
- Create a class website for app research. Throughout the semester, require students to post links to topics such as app standards, public safety, efficacy evidence, journals that publish mental health app research, sources of continuing education such as podcasts, webinars and conferences, app creator links, and app development and design information.
- Select a research study about a new mental health app, and create a flow chart depicting the design, development, testing, and research process that was used in the development of the app. Critique the research design and process.

Substance abuse

- Create a table in Google Docs for students to share links to evidence-based substance abuse apps.
- Write and present a report on the efficacy evidence of a substance abuse app that is designed for self-monitoring and relapse prevention.

### Disseminate Information During Clinical Staff Meetings

Staff meetings are an appropriate venue for discussing mental health mobile apps. Staff members may regularly present research evidence, app strengths and limitations, and suggestions for integrating particular apps into clinical practice. It will be useful to create an online document that permits staff to add and edit app information and provide links to research evidence. Staff members may also be invited to discuss experiences with using apps to support treatment.

### Integrate Apps Into Therapy

In order to integrate mental health mobile apps into therapy, it is necessary to collect information regarding patients’ mobile phones and their willingness to download and use mental health mobile apps. Relevant questions may be incorporated into intake forms. It is important to not give patients the impression that owning a mobile phone or a data plan is a requirement for therapy. Also, not all mobile phones have app capabilities, there may not be enough memory on some mobile phones to add apps, data plans may be limited, and some people may have privacy concerns related to storing sensitive data on their phones. When patients desire to use apps, practitioners must then make informed decisions about recommending appropriate apps. This decision making process is similar to that used in recommending books, workbooks, or other homework assignments.

The goal in recommending apps to individual clients is *purposeful alignment* of evidence-based apps with client needs and abilities. In order to best align particular apps with particular clients, professionals need to first interact with and test the apps themselves. One method of testing an app is to fictitiously assume symptoms of a related condition or mental disorder, while thinking through that app’s alignment with research and theory, ethics and privacy issues, relevance, benefits, consumer ease of use, engagement factors, and price value [9]. Apps need to be aligned with treatment goals, cultural backgrounds, and cognitive abilities. Practitioners may assign appropriate app activities such as homework, and then follow-up in subsequent visits. For example, if a patient has been directed to use a mood tracker app to monitor medication effectiveness, at subsequent sessions, the practitioner may review the data collected to assist with medication decisions.

Practitioners may also conduct surveys to monitor patient attitudes and effectiveness of mental health mobile apps. One method of monitoring patient attitudes is discussed by McGillicuddy et al [30]. After a demonstration of a prototype mobile phone-based monitoring system, renal transplant recipients completed a 10-item questionnaire regarding perceptions of the technology. They also completed a perceived stress scale and a medication adherence scale. Data collected through similar types of questionnaires will assist practitioners in making informed decisions about clinically integrating mental health mobile apps.

### Professional Association Conferences and Publications

The content analysis conducted by the authors of this article indicated the need for more technology-related topics at mental health professional conferences since technology-related and mental health mobile app topics averaged only 4% and 1%, respectively. While some professional association conferences offer technology-related sessions in technology tracks, reflecting an effort to include training in emerging technologies, grouping technology training into one track may delay the innovation diffusion process. Designated technology tracks tend to draw professionals who are innovators or early technology adopters and may fail to entice professionals in need of increased awareness and increased comfort levels with respect to mental health mobile apps and other technologies. Infusing emerging technologies across and within other tracks is likely to better assist in the diffusion process. As such, presenters need to share evidence-based technologies**,** provide demonstrations, include hands-on interaction, and deliver concrete suggestions for incorporating technologies into educational training and clinical practice.

### App Developers

Mental health mobile app developers need to consult with mental health experts to ensure that apps are based on sound psychological theory. Developers need to follow research protocols in producing app efficacy evidence. Attention to relevance and benefits [31], along with ethical, privacy, and security issues must guide app development. In addition, app developers need to create apps that will accentuate practitioner-patient collaborations in order to impel mHealth diffusion within the new health care paradigm [10].

## Conclusions

Evidence-based mental health mobile apps are relevant and beneficial for psychoeducation, patient empowerment, and for helping patients reach therapeutic goals. However, training on how to incorporate mental health mobile apps is lagging behind the rapid development of these mHealth technologies. As key influencers, counselor educators and professional associations have an ethical responsibility to learn about and to educate students and practitioners. To avoid or delay infusing mental health mobile apps into training and practice may even be considered by some to be professionally irresponsible. If the key leaders in the counseling field impede the diffusion of mental health mobile apps, patients and practitioners will be hindered from reaping the benefits of these technologies. Therefore, infusing mental health mobile apps into education and practice will help bring about diffusion of these innovations throughout the mental health social system.

## Abbreviations

eHealth: electronic health
mHealth: mobile health
REBT: rational emotive behavior therapy
UTAUT: Unified Theory of Acceptance and Use of Technology
UTAUT2: Unified Theory of Acceptance and Use of Technology-2
WHO: World Health Organization
